# Core design principles for nurturing organization-level selection

**DOI:** 10.1038/s41598-020-70632-8

**Published:** 2020-08-19

**Authors:** David Sloan Wilson, Melvin M. Philip, Ian F. MacDonald, Paul W. B. Atkins, Kevin M. Kniffin

**Affiliations:** 1 Department of Biological Sciences, Binghamton University, 4400 Vestal Parkway East, Binghamton, NY 13902-6000 USA; 2 Crawford School of Public Policy, Australian National University, Canberra, Australia; 3 Dyson School of Applied Economics and Management, SC Johnson College of Business, Cornell University, Ithaca, NY, USA

**Keywords:** Social evolution, Evolutionary theory

## Abstract

Dynamic relationships between individuals and groups have been a focus for evolutionary theorists and modelers for decades. Among evolutionists, selfish gene theory promotes reductionist approaches while multilevel selection theory encourages a context-sensitive approach that appreciates that individuals and groups can both matter. Among economists, a comparable contrast is found wherein the reductionist shareholder primacy theory most associated with Nobel laureate Milton Friedman is very different from the context-sensitive focus on managing common resources that Nobel laureate Elinor Ostrom pioneered. In this article, we examine whether the core design principles that Ostrom advanced can cultivate selection at supra-individual levels across different domains. We show that Ostrom’s design principles that were forged in the context of managing natural resources are associated with positive outcomes for human social groups across a variety of functional domains.

## Introduction

Evolutionary theorists have long debated whether groups are important units of selection or merely epiphenomenal artifacts of convergent lower-level interests. On the one hand, “selfish gene theory” has advanced the reductionist perspective that selection at the level of genes accounts for evolutionary change^1,2^. On the other hand, multilevel selection theory has proposed an integrative framework that appreciates that genes do serve as important measures of evolutionary change but that selection happens at a range of levels, including individuals, groups, and, at times, groups of groups^3–5^. While evolutionary theorists continue to debate which framework is most elegant and accurate for understanding evolutionary change, the contrast in approaches and assumptions is clear.

Economics, as a discipline, offers a similar divergence of frameworks in the work of two Nobel laureates: Milton Friedman and Elinor Ostrom. Comparable in some respects to selfish gene theory, Friedman famously proposed that the pursuit of narrow self-interests is what drives business success, writing that the only social responsibility of a business is to maximize profits for its shareholders within the limits of the law^6^. In this view, individuals in a free market who maximize their utilities (and, at a higher level, companies that maximize their profits) will be led, as if by an invisible hand, to benefit the common good^7^.

Friedman’s school of thought had a tremendous influence on economic policy, the business school curriculum, and the practices of business corporations around the world. Until very recently, it has been the guiding principle of the Business Roundtable, a group of CEOs of major US-based corporations. Viewed in retrospect, however, it stands upon a shaky theoretical foundation. Indeed, the actual observed consequences of profit maximization in a free market are different from what the metaphor of the invisible hand leads us to expect (i.e., the pursuit of narrow or low-level self-interests does not uniformly lead to the development of higher-level common goods)^8^.

Comparable in other respects to multilevel selection theory, Ostrom provided an alternative view^9–11^ that is more attentive to the importance of local context. She studied the famous “tragedy of the commons”—the tendency of groups to overexploit common-pool resources such as forests, pastures, fisheries, and ground water^12^. Orthodox economic thinking held that the only way to avoid the tragedy of the commons was to privatize the resource or impose top-down regulation. Employing a combination of empirical studies and game theory models, Ostrom showed that groups were capable of managing their common-pool resources on their own, but only if they implemented eight core design principles (CDPs) as introduced in this article via Table 1. Subsequent research has validated this result for common-pool resource groups^13^.

**Table 1:** Core design principles (CDPs) for the efficacy of groups with related functions.

	Core design principles (CDPs)	Function
1	Strong Group Identity and Sense of Purpose	Defines Group
2	Fair distribution of costs and benefits	Ensures effectiveness by balancing individual and collective interests
3	Fair and inclusive decision making	
4	Monitoring agreed upon behaviors	
5	Graduated sanctions for misbehaviors	
6	Fast and fair conflict resolution	
7	Authority to self-govern	Supports performance and engagement
8	Appropriate relations with other groups	Scales to Whole Systems

More recently, Wilson, Ostrom, and Cox^14^ articulated how the CDPs that were derived in relation to managing natural resources can be generalized from a multilevel evolutionary perspective to include virtually all groups whose members are working together to achieve a common goal. In this respect, the CDPs are mechanisms that facilitate the coordination of cooperative behaviors and the suppression of disruptive self-serving behaviors. Put another way, cooperation is itself a common-pool resource that can be supplemented or depleted. In the context of debates among evolutionary theorists: when CDPs are functioning within groups (in support of cooperative behaviors), then we would expect selection to be more likely at the group-level rather than self-serving individual levels.

The approaches advanced by Ostrom and Friedman provide a clear paradigmatic contrast. In the current research, we asked participants to provide information on a place of employment that they know well (described as a work group) compared to any other kind of group of their choice that they also know well (described as a non-work group). Focusing here on the relevance of CDPs for work groups compared to non-work groups, we (i) examine whether business groups tend to implement the CDPs less frequently than non-business groups and (ii) whether CDPs across all groups tends to be associated with better outcomes. Our expectations are that—consistent with Friedman’s perspective—CDPs will be less prevalent in business groups and that—consistent with Ostrom’s perspective—CDPs will be correlated in all types of groups with positive outcomes.

It is notable in the context of debates among evolutionary theorists that the CDPs offer a set of mechanisms that one would expect should (if present in an organization) be associated with the formation and success of supraindividual organizations. While prior writing on multilevel selection theory has focused on the importance of shared fate and phenotypic uniformity within groups as critical for selection to be expected at a given organizational level^3^, the CDPs offer a more specific and finer-grained array of mechanisms. A common feature of the CDP framework as well as multilevel selection theory is that they each offer value for understanding organizations across a wide array of domains. For example, Kniffin and Wilson^15^ examine airport ground-control units, rowing teams, and ranching communities through the lens of multilevel selection theory to showcase ways in which organizational rules and/or norms can be structured in support of supraindividual cooperation. The same analyses could have been done with greater precision or granularity through the CDP framework.

## Results

### Differences in CDP implementation

**Figure 1:**
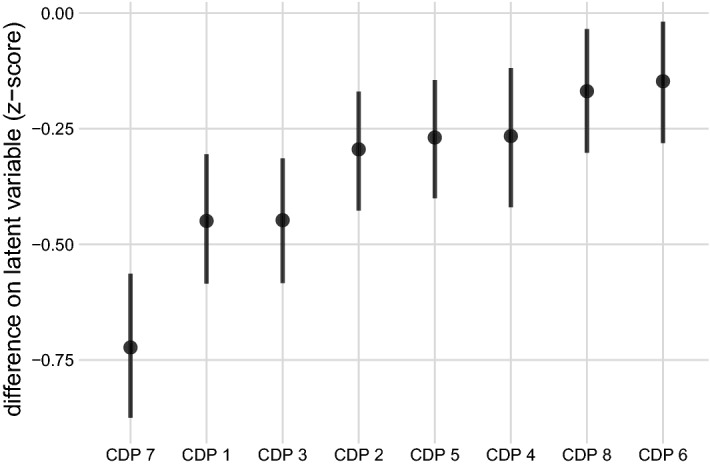
Mean differences in latent CDP scores between work and non-work groups, one model per outcome of interest*. Note*: Fig. 1 displays that work groups rated lower on perceived Core Design Principle (CDP) implementation than non-work groups. Plot displays estimated coefficients from eight multilevel ordinal models regressing CDP rating on group type (reference category = “non-work”; link function = probit). For each core design principle, the estimated rating difference for work groups, on the latent metric, is shown. Models were fit using a Bayesian framework, with circles and bars representing the Bayesian point estimate (median) and 95% credible interval of the posterior distribution, respectively.

Our first prediction is that work groups on average score lower on implementation of the CDPs than non-work groups. As shown in Fig. 1, which ranks the CDPs in order of the deficit for work groups, this prediction was confirmed. When compared with non-work groups, work groups are deficient in all of the CDPs, with the largest deficits for CDP7 (local autonomy), CDP1 (Strong sense of identity and purpose), and CDP3 (fair and inclusive decision-making). In other words, many employees feel that they do not have the autonomy to do their jobs as they see fit, do not strongly identify with the purpose of their place of employment, and do not feel part of the decision-making process.

Our second prediction is that the CDPs will be positively associated with important outcomes for both work and non-work groups. The results from multilevel regression models (reported in Table 2) reveal strong support for this in relation to Group Commitment, Group Cooperation, Psychological Needs Satisfaction, Group Satisfaction, and Group Trust. In other words, better implementation of the CDPs was positively associated with each of the organizationally beneficial outcomes across both work and non-work groups.

**Table 2:** Summary of mixed effects models, one per outcome of interest, showing relationship between CDPs, group type and outcomes.

	Commitment	Cooperation	Needs	Satisfaction	Trust
Predictors	Estimates CI (95%)	Estimates CI (95%)	Estimates CI (95%)	Estimates CI (95%)	Estimates CI (95%)
Intercept	0.05 (− 0.16, 0.28)	0.02 (− 0.12, 0.17)	0.04 (− 0.16, 0.25)	− 0.00 (− 0.12, 0.11)	0.03 (− 0.12, 0.20)
Work group	− 0.30 (− 0.39, − 0.21)	− 0.02 (− 0.10, 0.07)	− 0.19 (− 0.27, − 0.10)	− 0.11 (− 0.19, − 0.02)	− 0.14 (− 0.23, − 0.06)
CDPs	0.63 (0.58, 0.68)	0.67 (0.62, 0.72)	0.71 (0.66, 0.75)	0.73 (0.68, 0.77)	0.68 (0.63, 0.72)
Work group * CDPs	0.15 (0.06, 0.25)	− 0.03 (− 0.12, 0.07)	0.01 (− 0.09, 0.10)	− 0.00 (− 0.09, 0.09)	0.05 (− 0.04, 0.14)
**Random effects**					
*σ*^*2*^	0.04	0.12	0.02	0.05	0.06
*τ*_*00*_	0.96	0.89	0.99	0.95	0.95
*N* subject	487	488	488	488	490
*N* sample	8	8	8	8	8
Observations	950	942	943	956	967
Marginal R^2^/conditional R^2^	0.465/0.517	0.436/0.562	0.525/0.550	0.543/0.594	0.481 / 0.543

Summarizes results from five Bayesian mixed effects models, one per outcome of interest. For each predictor and interaction term, the Bayesian point estimate (median) and 95% credible interval of the posterior distribution is shown.

**Figure 2:**
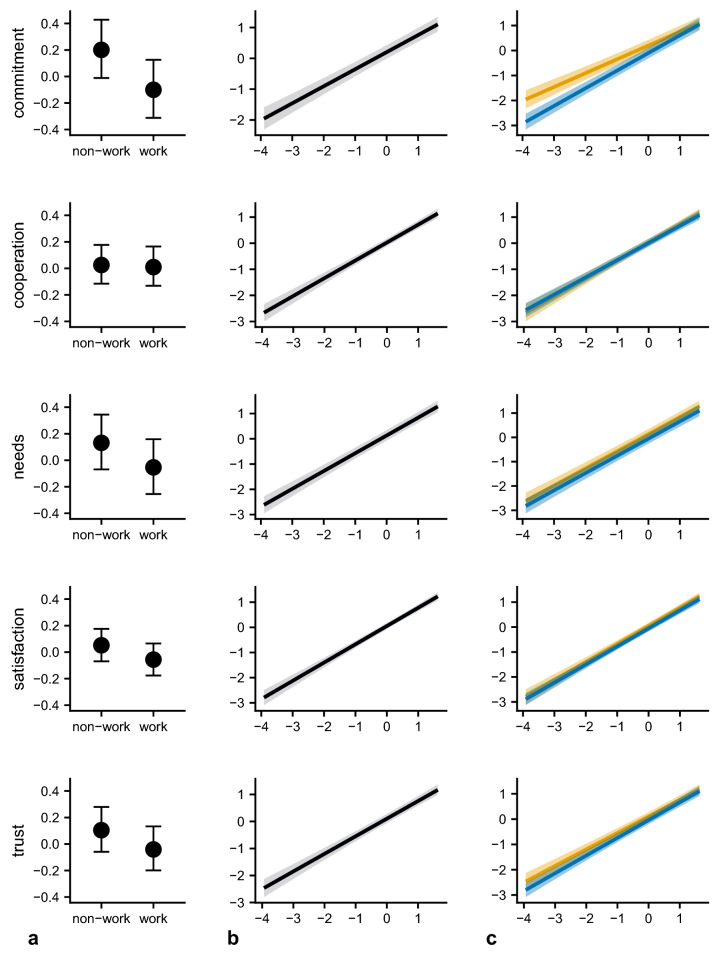
Conditional effects plots from five linear mixed effects models, one model per outcome of interest. *Note*: Fig. 2 displays the conditional effects of each predictor, with remaining held at the sample mean, from five Bayesian multilevel linear models (rows, one per outcome of interest). Column “a” shows the main effect of group type (estimated mean and 95% uncertainty interval [shaded]); “b” the main effect of composite CDP score, and “c” the interaction effect (Group Type * CDPs; blue = work, yellow = non-work [green = overlap]).

In addition to its primary illustration of the value of the CDPs for positive outcomes across groups with a wide range of missions, Fig. 2 (panel a) also shows a smaller “work group” effect. For four of the five group outcomes that we measured and describe in the Materials and Methods section, work groups were rated lower than non-work groups after accounting for differences in the CDPs; and, this is most pronounced for Group Commitment, where work groups score ~ 0.3 SD lower than non-work groups. When considering the full sample of both work and non-work group ratings, Fig. 2 (panel b) shows consistent positive relationships between the CDPs and the five outcomes. Further, for four of the five group outcomes, there was little evidence of an interaction effect between the work/non-work group distinction and implementation of the CDPs on outcome scores (Fig. 2, panel c). In other words, the relationship between overall implementation of the CDPs to group outcomes appears similar across group type. The single exception was Group Commitment, with the interaction suggesting that the overall CDP score is more strongly associated with commitment in work than non-work groups.

## Discussion

Our results strongly imply that places of employment (work groups) would benefit from the same CDPs as other kinds of groups and, as a class, tend to do a poorer job implementing them. Of course, these statements about average differences are compatible with the fact that some work groups function spectacularly well and other types of groups can spectacularly fail. Our study suggests, though, a positive relationship between the CDPs and organizational outcomes that is robust across work and non-work groups.

With respect to limits of the current study, all survey research must be supplemented with information from other sources to validate results. One potential bias of the self-report data that we examined relates to the potentially greater latitude that respondents have with respect to which non-work groups they rated. Specifically, if respondents had substantially more non-work groups to potentially rate, it is possible that the higher ratings reflect a higher possibility that one such group (for each respondent) functioned better. On the other hand, our prompt did not ask respondents to rate a successful or functional group per se; consequently, future research should address this potential bias. Additionally, a more comprehensive study would gather objective information about performance variables as well as group-specific implementation of the CDPs. Longitudinal studies that measure the long-term survival of groups (e.g., firms) would also be instructive. Finally, explicit attempts to improve implementation of the CDPs and monitor change in performance could establish the causal relationship implied by the strong correlations in our study.

We anticipate that future studies are likely to validate our results for two reasons. First, while the assumptions behind Friedman’s invisible hand perspective have been broadly criticized for being unrealistic^14^, Ostrom’s CDP approach rests on a strong theoretical foundation concerning the evolutionary dynamics of cooperation across species and, more specifically, our evolutionary history as a highly cooperative species. Second, while Ostrom’s CDP framework is not typically considered by business researchers, a great deal of supporting evidence already exists for the efficacy of the CDPs using different labels and typically in isolation from each other rather than as part of a holistic framework. In fact, entire review articles and meta-analyses document important and positive relationships between group performance (including outcomes such as profitability and survival) and a strong group identity and purpose (CDP1)^16,17^; equitable distribution of benefits (CDP2)^18^; fair and inclusive decision-making (CDP3)^19^; effective monitoring (CDP4)^20^; graduated sanctions for transgressions from agreed behaviors (CDP5)^21^; fast and fair conflict resolution (CDP6)^22^; and, allowing groups to manage themselves without excessive interference from outside the group (CDP7)^23^. Similarly, studies are starting to show that the same principles needed to govern interactions within groups are also needed to govern interactions between groups (CDP8)^24,25^.

In light of our own findings as well as prior research showing positive relationships between the CDPs and organizational performance, it is important to consider why the CDPs are not adopted more spontaneously or emerge more strongly from between-group competition. This question needs to be asked for all kinds of groups (including the common-pool resource groups studied by Ostrom) but especially business groups, where between-group competition is often intense. While it is outside of the scope of this study, we expect that failures of implementation are likely to have many causes. As Tolstoy wrote: “All happy families are alike; each unhappy family is unhappy in its own way.” One factor we can highlight for future research to consider is that individuals who gain at the expense of their groups do not easily relinquish their advantage, especially when they are among the elites and can successfully resist change.

An important and useful feature of the CDP framework is its scale-independence. The CDPs are needed to govern relations among groups in a multigroup population as well as among individuals within groups. This means that a company that cares only about maximizing profits for its shareholders is comparable—at a higher organizational level—to a narcissistic individual who puts his or her own interests above everyone else’s interests. By adopting and applying the stakeholder-focused CDP framework rather than the reductionist shareholder-primacy framework advanced by Friedman, the need for companies to become solid citizens that hold each other accountable for contributing to the welfare of society becomes clear. In this respect, the CDP framework offers a toolbox for organizational leaders to engage in the kind of moral behavior that social physicists have identified as a grand challenge facing researchers^26^. Indeed, CDP8, in particular, is most directly tied to ensuring that effective organizations are also contributing to greater goods beyond simply their own narrow ends.

Einstein wrote: “The theory decides what can be observed.” He meant that nothing is obvious or obscure all by itself, but only against the background of other assumptions and beliefs. For over half a century, the prominence of shareholder primacy theory has made it seem as if the lower-level pursuit of self-interest robustly benefits the common good. In contrast, the context-sensitive CDP framework that we have highlighted in the current study shows how individuals and institutions can effectively coordinate joint action and suppress disruptive self-serving behaviors. Given the scale-independence of the CDPs, it is notable that while we focused on the common resource of given organizations, the same framework can be applied at higher supra-firm levels in support of vibrant and sustainable economies at the global scale.

## Materials and methods

We created a 10-min online survey that asks participants to provide information on two groups that they know well as a member: A work group (defined as a place of employment) and any other kind of (non-work) group. The most common non-work groups that respondents rated were friend-groups (27.5%), family-groups (22.5%), sport-based groups (15.1%) community groups (14.7%), school-based groups (5.9%) and religious groups (3.1%) with the rest of the responses either unspecified (3.1%) or missing (8.2%). The most common work groups that respondents rated were for-profit businesses (66.1%), not-for-profit businesses (16.7%), and government agencies (9.2%) with the rest of the responses either unspecified (6.5%) or missing (1.4%).

The order of the two rating scenarios within the survey was randomized. The survey gathered demographic information about the participant (age, gender, geographical location) and the groups upon which they were reporting (location, size, time spent with the group). A classification of both work groups and non-work groups was provided. Next, participants rated the groups on five group outcome scales related to low turnover and greater engagement within groups: (1) Group Commitment; (2) Group Cooperation; (3) Psychological Needs Satisfaction; (4) Group Satisfaction; and, (5) Group Trust. We employed these measures since commitment, satisfaction, trust and whether or not the group meets basic psychological needs (e.g., a sense of belonging and autonomy) are known to determine the likelihood of someone staying, and also engaging, with the group. Finally, participants rated the degree to which the group implemented the eight core design principles (CDPs). See supplementary material for full information about these survey scales.

We collected data with informed consent of participants through efforts that sampled two sets of undergraduate students (n_1_ = 120, n_2_ = 40), three sets of M-Turk respondents (n_3_ = 54, n_4_ = 20, n_5_ = 148), business school students (n_6_ = 46), and participants employed in public and private sectors who were recruited using workshop listservs (n_7_ = 18, n_8_ = 44); and, our methods were performed in accordance with the relevant guidelines and regulations. More specifically, the research was conducted with approval of the Binghamton University Institutional Review Board. For our student populations, participants were compensated with course credit, and those who did not participate were allowed to complete another assignment to earn credit. For our M-Turk respondents, we paid participants $0.50 USD. Participants received no other forms of compensation. Once careless response patterns (e.g. 1,1,1,1,1,1,1,1,1,1,1), nonsensical cases (e.g. group size < 2), and those individuals who only supplied ratings under one scenario (e.g. “work” only) were removed, the total number of participants was 490 (varying slightly by analysis due to missing data).

## Supplementary information

Supplementary Information.
