# Supplementary material for: Core design principles for nurturing organization-level selection
Source: Sci Rep. 2020 Aug 19;10:13989. doi: 10.1038/s41598-020-70632-8 (PMC7438491; doi:10.1038/s41598-020-70632-8)
Supplement: Supplementary file 1 — Supplementary Information. [file 41598_2020_70632_MOESM1_ESM.pdf]

SUPPORTING INFORMATION (SI) TEXT

Core Design Principles for Nurturing Organization-Level Selection

David Sloan Wilson, Melvin M. Philip, Ian F. MacDonald, Paul W. B. Atkins,

Kevin M. Kniffin

Measures

The survey was comprised of several scales that are outlined below. For all scale items, participants indicated their agreement with each item using a 7pt Likert scale, ranging from strongly disagree to strongly agree. Individuals were scored on each scale by averaging responses to relevant indicators.

Core Design Principles (CDP) implementation was assessed using an eight-item scale developed by the researchers, with one statement corresponding to each design principle. The items are listed below. Scale reliability was good ($alpha_{work}$ = .87, $alpha_{non\text{-}work}$ = .85).

This group has a shared sense of identity and purpose

The demands and benefits of participating in this group are distributed equitably between its members.

Group members are included in the making of decisions about how the group should run.

Group members know what others in the group are doing.

If someone behaves in a way that is unhelpful or disruptive in this group, people respond appropriately to discourage that behavior.

There is fast and fair conflict resolution in this group.

This group has authority to govern itself without excessive interference from outside the group.

This group has collaborative relations with other groups.

Group Commitment was assessed using the four-item team commitment scale from (1). The items are listed below. Scale reliability was excellent ($alpha_{work}$ = .91, $alpha_{non\text{-}work}$ = .90).

I feel proud to belong to this group

I feel very committed to this group.

I am glad I belong to this group and not to another group.

I am willing to exert extra effort to help this group succeed.

Group Cooperation was assessed using the cooperative behaviors subscale from (2), consisting of six statements. The items are listed below. Scale reliability was acceptable ($alpha_{work}$ = .75, $alpha_{non-work}$ = .75).

In this group we work in a climate of cooperation.

In this group we discuss and deal with issues or problems openly.

While making a decision we take each other's opinion into consideration.

Some people hold back relevant information in this group. (R)

In this group people minimize what they tell about themselves. (R)

Most people in this group are open to advice and help from others.

Psychological Needs Satisfaction was assessed using six items from the basic psychological needs scale in (3). The items are listed below. Scale reliability was good ($alpha_{work}$ = .86, $alpha_{non-work}$ = .88).

I feel a sense of choice and freedom in the things I undertake in the group.

I feel that my voice and ideas are respected in the group.

I feel closely connected with the members of my group.

I feel connected with people that care for me, and for whom I care in this group.

I feel capable at what I do in this group.

I feel competent to achieve my goals in this group.

Group Satisfaction was assessed using the three-item team satisfaction scale from (1). The items are listed below. Scale reliability was good ($alpha_{work}$ = .89, $alpha_{non\text{-}work}$ = .89).

I am pleased with the way my colleagues and I work together.

I am satisfied with my present colleagues.

I am very satisfied with working in this group.

Group Trust was assessed using two items from the propensity to trust subscale combined with two from the perceived trustworthiness subscale in (2). The items are listed below. The lone reverse-coded item performed poorly and was dropped from the scale. Reliability was good ($alpha_{work}$ = .89, $alpha_{non\text{-}work}$ = .86).

Most people in this group do not hesitate to help a person in need.

The typical person in this group is sincerely concerned about the problems of others.

In this group people can rely on each other.

There are some hidden agendas in this group. (R)

Analysis

Differences in CDP Implementation

Perceived implementation of each CDP was assessed via agreement with a single statement using a 7pt Likert scale; e.g. "*This group has a shared sense of identity and purpose*" (CDP1). To accommodate the ordinal nature of responses and two levels of response clustering (in subjects and samples), multilevel ordinal models were used to test the prediction that work groups, on average, would score lower on implementation of the CDPs than non-work groups. Separate models were fit for each of the eight CDPs, in which ratings were regressed on the binary predictor "group type" (reference category = non-work; dummy coded), with random intercepts for subject and sample, and unequal variances in ratings allowed across scenario. Cases with missing data were excluded from analyses. For all models, the probit link function was used, reflecting the assumption that ratings represented categorization of a normally distributed latent trait.

All models were estimated within a Bayesian framework using the brms (Bayesian regression models using 'Stan') package for R (4-6). Weakly informative priors were assigned to all estimated model parameters: standard deviations, constrained to be positive, modelled using the half-student *t* distribution (3,0,10); intercepts using the student *t* (3,0,10), and regression coefficients using normal (0,3). Parameter estimates utilized four MCMC sampling chains, each with 4000 iterations of which the first 2000 were warmups to calibrate the sampler. For all models, no warnings were returned during sampling and model estimation. $\hat{R}$ values for

parameter estimates were equal to 1.00, indicating all four chains converged to a common distribution. This was confirmed via visual inspection of MCMC trace plots.

Relationship between CDPs, Group Type and Outcomes

For each outcome metric, composite scores were calculated using the average of participant's responses to scale indicators. Similarly, an aggregate 'perceived CDP' score was created by averaging participants responses to the 8 CDP items (work alpha = .87; non-work alpha = .85). To examine the relationship between CDP implementation, group type and outcomes, five multilevel linear models were fitted (one per outcome). In each, the outcome of interest was regressed on overall CDPs score, group type, and the interaction between CDPs and group type. All models included random intercepts for subject and sample, with both outcomes and CDP scores standardized (*z*-scores). Sum contrasts were used for group type (non-work = -.5, work = +.5). Initial exploration (correlations, *t*-tests) and model testing did not warrant inclusion of additional covariates such as gender, group size and hours per week spent on group activities, so the decision was made to stick with the simpler models to aid interpretation. Models with and without additional covariates did not differ in their predictive accuracy, as all estimated model differences in eldp_loo were less than twice the standard error of the estimated difference itself (7).

Again, all models were estimated within a Bayesian framework using the 'brms' package. For priors, a normal distribution (0,3) was assigned to estimated regression coefficients, intercepts and standard deviations (constrained to be positive). Parameter estimates utilized four MCMC

sampling chains, each with 6000 iterations of which the first 3000 were warmups to calibrate the sampler. Again, no model warnings were returned and all $\hat{R}$ values for parameter estimates were equal to 1.

References

1. Van der Vegt, G., Emans, B. & Van De Vliert, E. Team members' affective responses to patterns of intragroup interdependence and job complexity. *J Manage*, **26**, 633-655 (2000).

2. Costa, A. C. & Anderson, N. R. Measuring trust in teams: Development and validation of a multifaceted measure of formative and reflective indicators of team trust. *Euro J Work Org Psych*, **20**, 119-154 (2011).

3. Chen B. et al. Basic psychological need satisfaction, need frustration, and need strength across four cultures. *Motiv Emot*, **39**, 216-236 (2015).

4. Bürkner, P. C. brms: An R package for Bayesian multilevel models using Stan. *J Stat Softw*, **80**; 10.18637/jss.v080.i01 (2017).

5. Carpenter B. et al. Stan: A probabilistic programming language. *J Stat Softw*, **76**; 10.18637/jss.v076.i01 (2017).

6. R Core Team. *R: A language and environment for statistical computing*. R Foundation for Statistical Computing. (Vienna Austria), https://www.R-project.org/ (2018).

7. Vehtari, A., Gelman. A. & Gabry, J. Practical Bayesian model evaluation using leave-one-out cross-validation and WAIC. *Stat comp*, **27**, 1413-1432 (2017).
